# Changes in the global burden of foreign body aspiration among under-5 children from 1990 to 2019

**DOI:** 10.3389/fped.2023.1235308

**Published:** 2023-09-01

**Authors:** Yuying Wu, Xin Zhang, Zaigang Lin, Chenyu Ding, Yuxuan Wu, Yue Chen, Desheng Wang, Xuehan Yi, Fa Chen

**Affiliations:** ^1^ Department of Epidemiology and Health Statistics, School of Public Health, Fujian Medical University, Fuzhou, China; ^2^ Fujian Branch of Shanghai Children’s Medical Center, College of Clinical Medicine for Obstetrics & Gynecology and Pediatrics, Fujian Children’s Hospital, Fujian Medical University, Fuzhou, China; ^3^ Laboratory Animal Center, Fujian Medical University, Fuzhou, China; ^4^ Department of Neurosurgery, Neurosurgery Research Institute, The First Affiliated Hospital, Fujian Medical University, Fuzhou, China; ^5^ Department of Otolaryngology Head and Neck Surgery, Fujian Medical University Union Hospital, Fuzhou, China; ^6^ Clinical Research Unit, The Second Affiliated Hospital, Fujian Medical University, Quanzhou, China

**Keywords:** foreign body aspiration, socio-demographic index, DALYs, under-5 children, global burden

## Abstract

**Background:**

To evaluate the changes in the global burden of foreign body aspiration (FBA) among children under 5 years old at regional, age, sex, and socio-demographic index (SDI) levels between 1990 and 2019.

**Methods:**

Data on FBA was derived from the Global Burden of Disease (GBD) Study 2019 database on pulmonary aspiration and foreign body in airway. The means and 95% uncertainty intervals (UIs) were calculated for incidence, and disability-adjusted life-years (DALYs). The temporal trends were represented by estimated annual percentage change (EAPC) using Joinpoint regression.

**Results:**

Globally, FBA caused 109.6 (95% UI: 69.5, 175.7) per 100,000 incidence and 317.9 (95% UI: 270.7, 372.4) per 100,000 DALYs under 5 years old in 2019. Many European countries (such as Italy, Netherlands, Iceland, etc.) showed a high incidence rate, but did not cause a large disease burden (DALYs all less than 200 per 100,000). Compared to 1990, although a decrease in both incidence and DALYs occurred in 2019, the Joinpoint regression showed an increasing trend in incidence rate from 2014 to 2019 [APC: both (2.10), female (2.25), male (1.98), *P *< 0.05)], especially China, Netherlands, and Malta. Despite the lower incidence rate in early neonatal group and middle SDI areas, they instead resulted in higher DALYs than other age groups and areas.

**Conclusion:**

Although declines occurred in incidence and DALYs of FBA among children under 5 years of age from 1990 to 2014, an upward trend began to emerge from 2014 to 2019. The incidence and DALY rates were correlated with age and SDI. Increased efforts are needed to improve the necessary monitoring and reporting systems, hazard assessment, and public education activities.

## 1. Introduction

Foreign body aspiration (FBA) remain a common public health concern in pediatrics globally, contributing to serious health consequences and economic burden, especially in preschool children (1–3). It has been reported that young children are particularly at risk for FBA, with nearly 98% of cases less than 5 years of age (4, 5). Compared with adults, children harbor smaller airways and greater airway resistance, rendering them more vulnerable to severe airflow obstruction. In addition, dental development and high mobility and distractibility during feeding further put them at risk (5–7). The most common causes mainly include food, coins, toys, and balloons, among which, organic foreign bodies were more common than inorganic (5, 6, 8–11).

Several studies have documented that inhalation of foreign bodies can result in acute or chronic complications, such as recurrent respiratory infections, chronic cough, persistent wheezing, pulmonary or segmental collapse and bronchiectasis (10). As for young children, FBA was reported to cause a persistent increase in pediatric cerebral palsy patients (12). Delayed diagnosis is an important contributing factor to complications. In many cases, choking episodes may be unattended, leading to a delay in diagnosis and potentially dangerous consequences for the patient's health and life, especially if there is no clear history and clinical presentation (6, 7, 9, 13). Additionally, information about the non-fatal health outcomes of foreign body aspiration is generally overlooked (11). Therefore, there is a growing imperative to focus on the disease burden caused by FBA among young children. Given the diverse living environment, lifestyle, cultural and economic situations of people in various regions, the disease burden may vary by geographical location. However, to date, few studies have systematically described the global burden of FBA among young children. In this study, we aimed to comprehensively understand the global, regional, and national incidence and disability-adjusted life years (DALYs) of FBA among children under 5-year-olds, from 1990 to 2019, by age, sex, and socio-demographic index (SDI).

## 2. Methods

### 2.1. Overview

Data on FBA was exacted from the Global Burden of Disease (GBD) Study 2019 via the Global Health Data Exchange website (http://ghdx.healthdata.org). In this study, FBA refers to pulmonary aspiration and foreign body in airway as defined in the GBD 2019. The GBD 2019 systematically assesses the health burden of 369 diseases and injuries from 1990 to 2019 including 204 countries and territories. Other studies have described a detailed methodology for estimating incidence and DALY rates (14). This study was conducted in accordance with the Helsinki Declaration of 1975. Based on publicly available dataset, this study did not require ethical approval. Informed consent was waived because GBD 2019 uses de-identified aggregated data.

### 2.2. Definition of foreign body aspiration

In GBD 2019, The International Classification of Diseases ninth and tenth revision (ICD-9 and ICD-10) codes used in the analyses for foreign body aspiration (i.e., pulmonary aspiration and foreign body in airway) are 770.1–770.18, E911-E912.09, E913.8-E913.99 and W75-W76.9, W78-W80.9, W83-W84.9.

### 2.3. Data processing and presentation

Joinpoint regression was used to assess the time trends and the annual percentage change (APC) in incidence and DALY rates in 1990–2019 for FBA among children under 5 years old. The analysis was then performed by 204 countries, 5 SDI regions, 21 GBD regions, different genders, and age groups. Temporal trends in rates (per 100,000 population) over a speciﬁc time interval, measured by the value of estimated annual percentage change (EAPC), were calculated using a generalized linear model. In the formula y = α + βx + *ε*, where y refers to ln (rate) while x means the calendar year. EAPC values were calculated using the formula EAPC = 100 × (e^β−1^). The rates were considered to be: (1) an upward trend if the estimated value of the EAPC and its lower 95% confidence intervals (CI) above zero; (2) a downward trend if the estimated value and its upper 95% CI below zero; (3) stable if the 95% CI contains zero. We also analyzed the correlation between SDI and incidence and DALY rates in 2019. Hierarchical clustering based on the EAPC of incidence and DALY rates in 2014–2019 divided the 204 countries into four categories (significant increase, significant decrease, remained stable or minor increase, minor decrease).

DALY is all healthy life years lost from morbidity to mortality, which is estimated as the sum of years of life lost (YLL) and years of life lived with disability (YLD). SDI was evaluated based on the total fertility rate among females younger than 25 years old, educational attainment for those aged 15 years or older, and lag distributed income per capita. The 204 countries and territories were then placed into 5 categories according to the SDI: low-SDI, low-middle-SDI, middle-SDI, high-middle-SDI, and high-SDI. Children under 5 years old are classified as early neonatal, late neonatal, post neonatal and 1 to 4 according to the existing age groups of GBD. Early neonatal is 0–6 days after birth. Late neonatal is 7–27 days and post neonatal is 28–364 days.

In GBD 2019, every estimate was calculated 1,000 times, and the final data was presented as the mean of these estimates. 95% uncertainty interval (UI) was calculated for each parameter in the analysis, which was determined using the 25th and 975th values of the ordered 1,000 draws. Joinpoint regression was performed using the Joinpoint software (version 4.7.0) developed by the Surveillance Research Program of the US National Cancer Institute. Other statistics and graphing were performed using R (version 4.1.0).

## 3. Results

### 3.1. Overall burden

**Figure 1:**
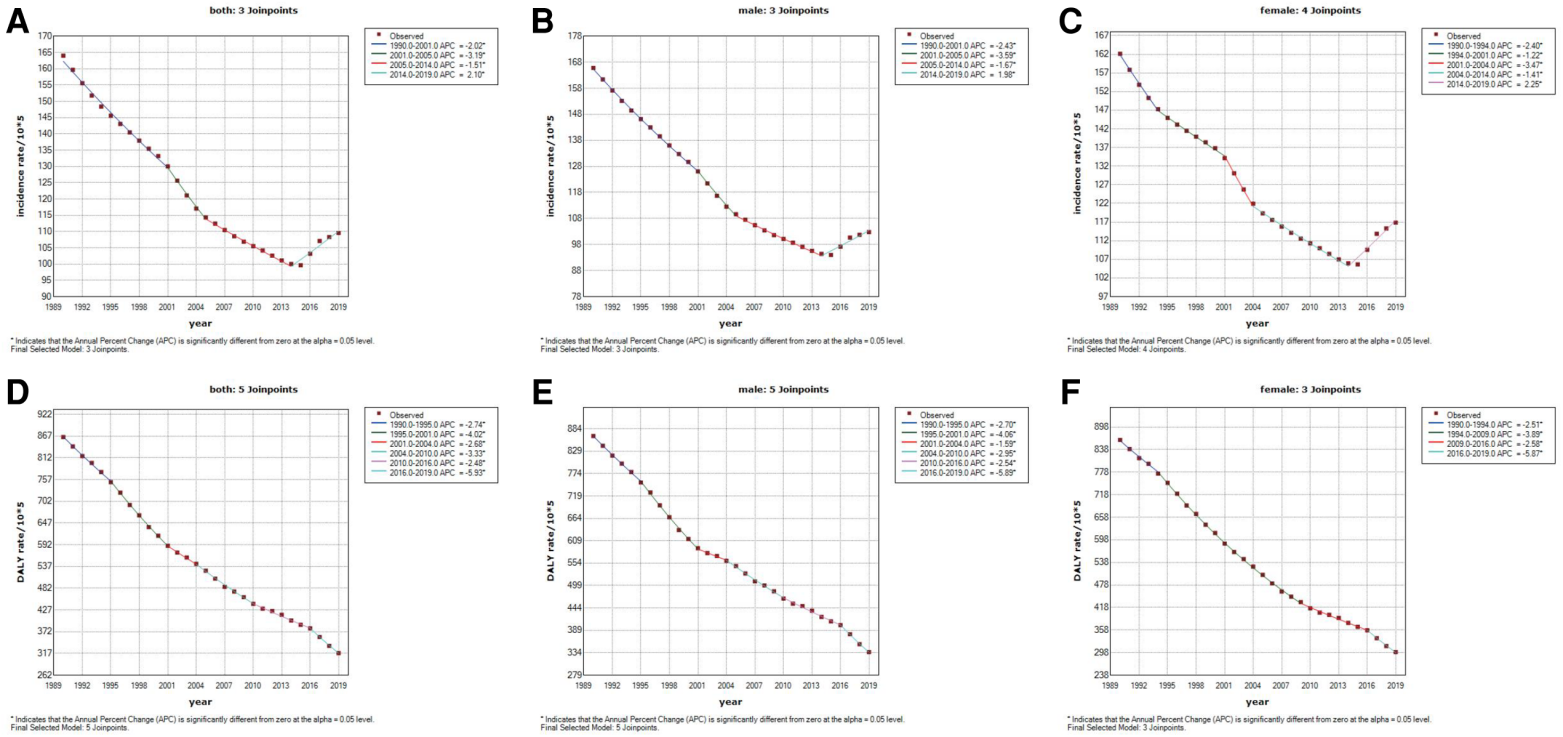
Temporal trends in the incidence and disability-adjusted life-year (DALY) rates of global foreign body aspiration among children under 5 years of age by sex from 1990 to 2019. (**A**) Incidence rate for both sexes; (**B**) Incidence rate for males; (**C**) Incidence rate for females; (**D**) DALY rate for both sexes; (**E**) DALY rate for males; (**F**) DALY rate for females.

Globally, FBA caused 109.6 (95% UI: 69.5, 175.7) per 100,000 incidence rate and 317.9 (95% UI: 270.7, 372.4) per 100,000 DALY rate under 5 years old in 2019 (Table 1). Overall, compared to 1990, a decrease in both the incidence rate and the DALY rate occurred in 2019. The EAPC from 1990 to 2019 were −1.7 (95% UI: −1.9, −1.5) for incidence rate, and −3.3 (95% UI: −3.3, −3.2) for DALYs, respectively. Of note, the Joinpoint regression revealed an increasing trend in the incidence rate of FBA in 2014–2019 [APC: both (2.10), female (2.25), male (1.98), *P* < 0.05)], although there was a significant decline in 1990–2014 (Figures 1A–C). The DALY rate presented a continuous downward trend, with an EAPC of −4.6 (95% UI: −5.4, −3.8) (Figures 1D–F).

**Table 1:** The incidence and DALY rates (per 100,000) of foreign body aspiration under 5 years old by region, gender and age in 1990, 2014 and 2019, and its temporal trends of 1990–2019 and 2014–2019.

	Incidence rate (per 100,000)					DALY rate (per 100,000)				
	1990 (95% UI)	2014 (95% UI)	2019 (95% UI)	EAPC (1990–2019) (95% UI)	EAPC (2014–2019) (95% UI)	1990 (95% UI)	2014 (95% UI)	2019 (95% UI)	EAPC (1990–2019) (95% UI)	EAPC (2014–2019) (95% UI)
Global	164 (105.4, 257.2)	100 (65.6, 154.2)	109.6 (69.5, 175.7)	−1.7 (−1.9, −1.5)	2.2 (1.6, 2.7)	864.9 (766.9, 981.5)	400.2 (357.7, 443.8)	317.9 (270.7, 372.4)	−3.3 (−3.3, −3.2)	−4.6 (−5.4, −3.8)
Sex										
Female	162.1 (103, 253.5)	105.9 (69.2, 163.7)	116.8 (73.9, 187.3)	−1.4 (−1.6, −1.2)	2.3 (1.7, 2.9)	863.6 (740.3, 990.2)	377.1 (335.9, 417.7)	299.2 (256.7, 348.3)	−3.6 (−3.6, −3.5)	−4.7 (−5.4, −3.9)
Male	165.8 (107.3, 259.4)	94.4 (62.1, 146.3)	102.7 (65.4, 164.4)	−2 (−2.2, −1.8)	2 (1.4, 2.6)	866.2 (715.1, 1025.9)	422 (370.1, 473.2)	335.5 (281.1, 397.5)	−3 (−3.1, −2.9)	−4.6 (−5.4, −3.7)
Age										
Early Neonatal	165.5 (108.6, 271.9)	85.5 (58.2, 132.5)	92 (58.3, 153.6)	−2.3 (−2.5, −2.1)	1.8 (1.1, 2.4)	16147.6 (14066.6, 18472.3)	9382 (7982.4, 10901.1)	8071.1 (6658.3, 9728.2)	−2.6 (−2.8, −2.5)	−3.1 (−3.6, −2.6)
Late Neonatal	168.4 (111.6, 275.6)	87.3 (60.4, 134.5)	93.7 (60, 153.3)	−2.3 (−2.5, −2.1)	1.7 (1.1, 2.3)	6158 (5447.1, 6993.7)	2764.1 (2413.1, 3140.3)	2276.6 (1927.5, 2664.4)	−3.4 (−3.6, −3.3)	−4 (−4.6, −3.3)
Post Neonatal	197.4 (124.8, 299.4)	108.3 (70.2, 160.7)	114 (70, 175.7)	−2.3 (−2.5, −2.1)	1.2 (0.8, 1.6)	2775.6 (2451.4, 3178.8)	1273.9 (1145.8, 1407.6)	1003.2 (851.6, 1174.1)	−3.3 (−3.4, −3.2)	−4.7 (−5.5, −4)
1 to 4	155.9 (96, 256.3)	98.3 (61.5, 159.1)	108.9 (66.5, 180.8)	−1.5 (−1.7, −1.3)	2.4 (1.8, 3)	240.8 (203, 286.8)	114.9 (97.8, 134.6)	95 (77.9, 114.1)	−3.1 (−3.1, −3)	−3.7 (−4.1, −3.3)
SDI region										
Low SDI	141.6 (85.3, 229)	102 (63.7, 160.5)	107.1 (64.7, 172.3)	−1.1 (−1.2, −1)	1.3 (0.7, 1.8)	471.3 (375.9, 575)	277 (225.8, 337.9)	239.8 (186.2, 308.9)	−2.2 (−2.2, −2.1)	−2.9 (−3.3, −2.5)
Low-middle SDI	154.6 (97, 243.6)	96 (60.9, 151.2)	101.7 (62, 165.3)	−1.7 (−1.9, −1.5)	1.4 (0.9, 2)	791.4 (675.2, 922)	397.9 (342.2, 455.4)	320.9 (265.9, 387.1)	−2.8 (−2.9, −2.8)	−4.3 (−4.8, −3.8)
Middle SDI	148.9 (92.7, 236.2)	78.2 (51.1, 122.8)	86.8 (54, 142.6)	−2.5 (−2.8, −2.2)	2.3 (1.8, 2.9)	1186.2 (1030, 1373.6)	504.6 (446.5, 565.9)	382.5 (316.9, 449.3)	−3.8 (−3.9, −3.7)	−5.5 (−6.7, −4.3)
High-middle SDI	211.2 (137.3, 327.3)	122.8 (83.7, 185.4)	137.9 (89.4, 217.5)	−1.9 (−2.1, −1.6)	2.7 (2, 3.5)	947.5 (844.8, 1077.9)	476.3 (438.2, 521.4)	358.6 (302.2, 419.6)	−3.1 (−3.3, −3)	−5.6 (−6.4, −4.8)
High SDI	197.3 (137.7, 288.5)	146.7 (102.1, 218.2)	178.7 (121.4, 268.3)	−0.4 (−0.8, −0.1)	4.5 (3.6, 5.3)	446 (419.9, 475.6)	304.9 (280.3, 325.5)	270.8 (242.3, 299.2)	−0.8 (−1.2, −0.4)	−2.7 (−3.7, −1.6)
21 GBD region										
Andean Latin America	726.4 (478.7, 1050.9)	213.2 (146.6, 311.8)	209.6 (135.4, 318.1)	−5 (−5.3, −4.7)	−0.1 (−0.5, 0.4)	9093.2 (7129.1, 11195.7)	1901.6 (1486.3, 2384.8)	1424.9 (983.1, 2014.1)	−7 (−7.4, −6.6)	−5.7 (−6.3, −5)
Australasia	151.6 (98.8, 241.8)	137 (94.3, 203.1)	147.7 (100.1, 222.7)	0 (−0.1, 0.2)	1.7 (1.3, 2.2)	223.5 (199.3, 250.3)	195.7 (170.1, 220.4)	154.6 (120.3, 192.5)	−0.5 (−1, 0)	−4.2 (−6, −2.3)
Caribbean	206.1 (134.4, 314.2)	137.3 (87, 213.8)	144.5 (89.5, 229.9)	−1.2 (−1.4, −1.1)	1.4 (0.7, 2.1)	2164.2 (1617.4, 2797.9)	1158.5 (758.7, 1651.8)	1064.4 (668.5, 1481)	−2 (−2.4, −1.5)	−1.9 (−2.4, −1.4)
Central Asia	311.7 (219, 459.5)	217.5 (154.7, 325.6)	234.4 (160.5, 353)	−1.2 (−1.3, −1.1)	1.9 (1.1, 2.8)	1080.9 (913.1, 1254.6)	656 (541.5, 799.6)	504.2 (386.4, 663.9)	−2.5 (−2.8, −2.1)	−5.2 (−5.4, −4.9)
Central Europe	283.8 (196, 426.3)	154.7 (105.2, 239.6)	167.3 (112.8, 263.5)	−1.9 (−2.1, −1.6)	2.2 (0.9, 3.6)	926.2 (847.6, 1032.6)	213.1 (196.2, 231.9)	178.8 (139.3, 227.3)	−5.3 (−5.6, −5.1)	−3.3 (−4, −2.6)
Central Latin America	242.3 (162.8, 366.9)	122.9 (82.9, 183.3)	132.2 (86.3, 204.7)	−2.8 (−2.9, −2.6)	1.2 (−0.8, 3.2)	1399.3 (1245.4, 1545.1)	869.3 (700.2, 1069.3)	706.4 (499.7, 962.4)	−2.2 (−2.5, −2)	−4.1 (−4.1, −4)
Central Sub-Saharan Africa	183 (103.2, 291.2)	125.6 (76.4, 197.6)	125.2 (74.4, 200.4)	−1.5 (−1.7, −1.3)	0.1 (−0.2, 0.4)	333.8 (199, 493.3)	167.5 (100, 272.7)	117 (68.9, 210)	−3.4 (−3.7, −3.1)	−7.1 (−7.8, −6.4)
East Asia	139 (75.1, 234.6)	48.9 (29.5, 82.1)	75 (41.8, 133.1)	−4.3 (−5.3, −3.2)	9.1 (8.3, 9.9)	1706.6 (1412.6, 2138.1)	862.1 (758.5, 971)	585.4 (479.2, 701.3)	−3.1 (−3.4, −2.8)	−7.6 (−9.9, −5.4)
Eastern Europe	254.5 (167.8, 388.2)	187.9 (124.7, 285.4)	190.7 (123.3, 296.8)	−1.3 (−1.5, −1)	0.6 (0.1, 1.2)	637.4 (590.7, 702.8)	590.4 (552.4, 636.1)	406.3 (324.7, 499.4)	−1.9 (−2.8, −1)	−7.3 (−9.3, −5.2)
Eastern Sub-Saharan Africa	130.6 (78.2, 213.1)	92.6 (57.6, 147.8)	96.4 (58, 157.7)	−1.2 (−1.3, −1.1)	1.1 (0.6, 1.6)	266.9 (189.3, 371.7)	156.8 (117.5, 206.4)	124.3 (90.3, 172)	−2.1 (−2.4, −1.8)	−4.5 (−4.6, −4.4)
High-income Asia Pacific	117.4 (75.1, 184.8)	67.9 (43.6, 107.3)	72.5 (45.5, 118.3)	−1.8 (−2, −1.6)	1.7 (0.9, 2.6)	753.1 (640.9, 870.2)	219.6 (200.2, 234.6)	192 (167.8, 217.4)	−4.6 (−5, −4.2)	−2.6 (−2.9, −2.3)
High-income North America	109.4 (70.5, 173.5)	104 (66.9, 162.4)	134 (84.4, 216.1)	0.1 (−0.2, 0.3)	5 (2.9, 7.1)	331.4 (309.6, 353.5)	548.2 (499.3, 595)	493.3 (447.5, 539.6)	2.6 (2.1, 3)	−2.5 (−3.8, −1.2)
North Africa and Middle East	99.1 (59.3, 160.8)	64.9 (39.3, 103.5)	69.7 (41.2, 115.8)	−1.5 (−1.6, −1.3)	1.8 (1, 2.6)	632.5 (470.2, 886.9)	279.6 (214.4, 373)	207 (154.8, 276.3)	−3.5 (−3.7, −3.4)	−6 (−6.3, −5.7)
Oceania	40 (22.7, 67.7)	37 (20.3, 62.6)	36.6 (19.4, 63.9)	−0.1 (−0.3, 0)	−0.1 (−0.4, 0.1)	142.9 (104.2, 297.1)	103.1 (72.1,261.5)	90.8 (59.1, 237.4)	−1.1 (−1.4, −0.8)	−2.4 (−2.6, −2.3)
South Asia	163.5 (101.4, 259.8)	104.9 (65.8, 168.5)	111.6 (67.1, 183.1)	−1.5 (−1.7, −1.3)	1.6 (0.9, 2.3)	413.4 (341.5, 492.2)	272.3 (224.8, 324.2)	229.9 (183.2, 286)	−1.8 (−1.9, −1.7)	−3.6 (−4.1, −3.2)
Southeast Asia	83.5 (48, 137.8)	46.5 (27.1, 76.1)	48.2 (27.9, 81.1)	−2.1 (−2.3, −1.9)	1.1 (0.3, 1.8)	639.4 (503.4, 831.5)	311.4 (255.8, 384.3)	262.8 (207.7, 335.5)	−3 (−3.2, −2.9)	−3.4 (−3.5, −3.3)
Southern Latin America	317.5 (227.9, 447.8)	126.8 (89.9, 182.1)	134.2 (90.8, 203.3)	−3.2 (−3.5, −2.9)	1.4 (1, 1.8)	2781.9 (2470, 3110.2)	565.2 (489.7, 663.6)	474.3 (349, 635.2)	−6.4 (−6.9, −5.9)	−3.4 (−4.5, −2.2)
Southern Sub-Saharan Africa	83.7 (52, 133)	71.9 (45.3, 114.3)	76.6 (47.8, 124.4)	−0.3 (−0.4, −0.2)	1.6 (1, 2.2)	449.9 (339.3, 569.7)	254.5 (195.8, 319.4)	238.8 (176.2, 321.1)	−1.6 (−2.3, −0.9)	−1.4 (−1.9, −0.9)
Tropical Latin America	169.7 (103.2, 277.7)	64.6 (36.9, 108.3)	77 (42.6, 137)	−2.7 (−3.6, −1.7)	3.1 (1, 5.1)	880.8 (759.2, 1076)	929.7 (763.1, 1113.2)	761 (598.3, 957.3)	−0.2 (−0.4, 0)	−3.9 (−4.5, −3.3)
Western Europe	394.1 (284.8, 559.7)	292.9 (208, 426.7)	326.6 (227.5, 480.3)	−0.3 (−0.6, −0.1)	2.9 (1.1, 4.8)	399.5 (368.8, 444.9)	96.1 (84.6, 104.2)	85.8 (72.1, 101.1)	−4.7 (−5.2, −4.3)	−2.5 (−3.5, −1.4)
Western Sub-Saharan Africa	149.3 (92.4, 236.3)	111 (70.4, 174.7)	120 (74, 191.7)	−0.9 (−1, −0.8)	2 (1.1, 2.8)	359.4 (241.4, 497.1)	248.4 (181.5, 339)	228.8 (162.9, 318.4)	−1.6 (−1.7, −1.5)	−1.6 (−2.8, −0.4)

DALY, disability-adjusted life-year; EAPC, estimated annual percentage change; SDI, socio-demographic index.

### 3.2. Regions

**Figure 2:**
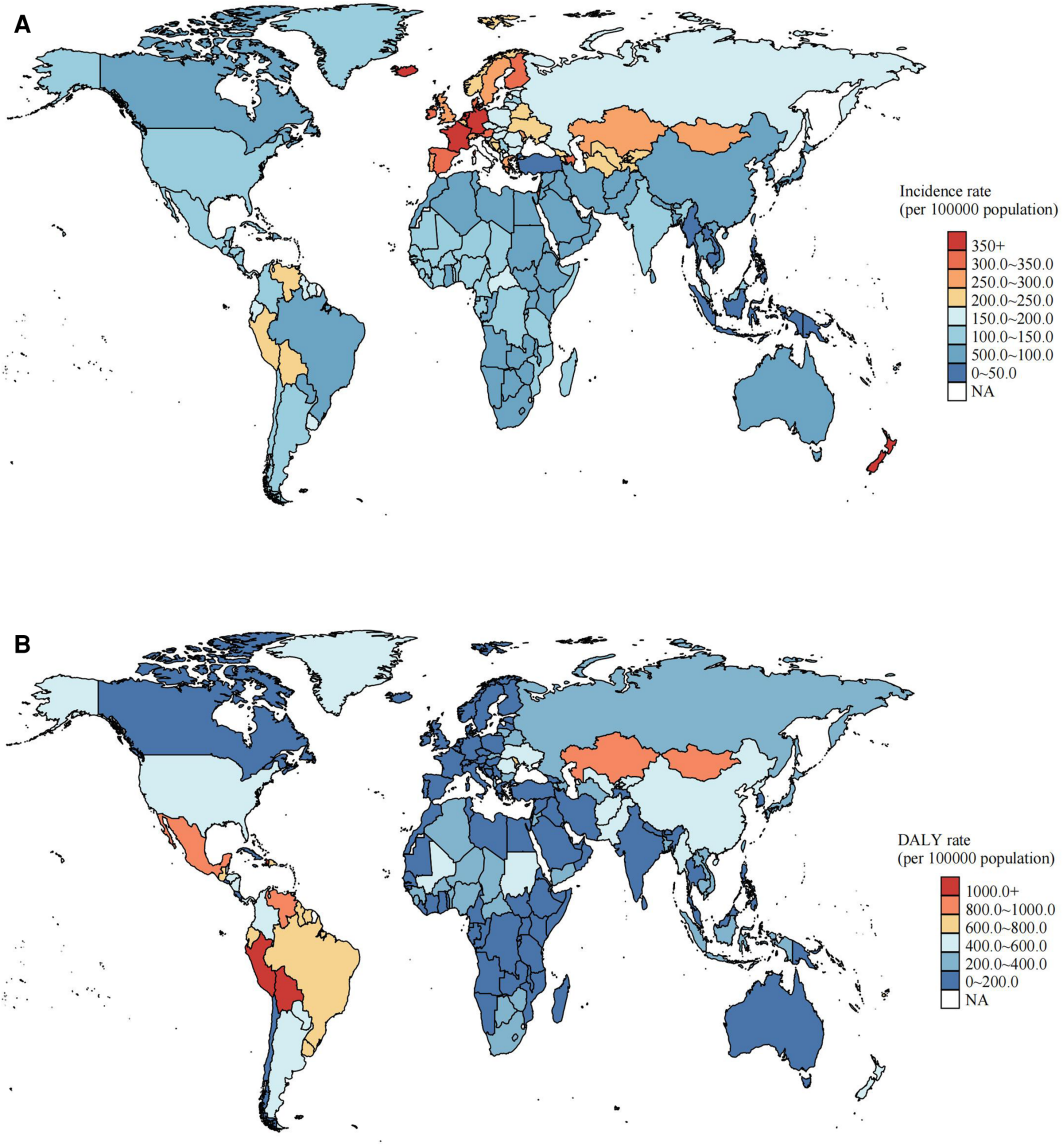
The incidence and disability-adjusted life-year (DALY) rates of foreign body aspiration among children under 5 years of age in 204 countries and territories in 2019. (**A**) Incidence rate; (**B**) DALY rate.

In 2019, Italy, New Zealand, Netherlands, Iceland, France, and Germany had higher incidence rates (all more than 350 per 100,000) and most of these countries are located in Europe. Many other European countries also showed a high incidence rate. Nevertheless, these countries did not cause a large disease burden (DALYs all less than 200 per 100,000, Figure 2). Conversely, Haiti, Mexico and Brazil did not have a high incidence rate but their DALY rates were in the top 10, especially Haiti, which had the first DALY rate [2028.2 (95% UI: 1111.6–3062.4)]. And the countries with the second and third highest DALY rates were Bolivia and Peru (both more than 1,500 per 100,000), respectively (Figure 2 and Supplementary Table S1). From 1990 to 2019, 204 countries and territories experienced a decline or less fluctuation in incidence rate. But from 2014 to 2019, most of the countries showed an increase in incidence rate, especially China, Netherlands, and Malta, whose EAPCs (2014–2019) were in the top three places, with 9.4 (95% UI: 8.5, 10.2), 7.5 (95% UI: 6.2, 8.9) and 7.2 (95% UI: 3.7, 10.7), respectively. It is noteworthy that the incidence rate in 2019 were nearly double compared to 2014 (75.6 vs. 48.7 per 100,000). In contrast, the DALY rates showed a decrease in most countries, but Mali, Dominica, Puerto Rico, Lesotho and Barbados were shown as rising (Supplementary Table S1). Hierarchical clustering of different countries according to EAPC (2014–2019) of incidence and DALY rates showed only Iran (Islamic Republic of) had a significant decrease. Other countries are detailed in the Supplementary Figure S1.

In 2019, in 21 GBD regions, Andean Latin America and Caribbean had high incidence rates [209.6 (95% UI: 135.4, 318.1) and 144.5 (95% UI: 89.5, 229.9)] and first and second highest DALY rates [1424.9 (95% UI: 983.1, 2014.1) and 1064.4 (95% UI: 668.5, 1481)]. East Asia and Tropical Latin America had low incidence rates [75 (95% UI: 41.8, 133.1) and 77 (95% UI: 42.6, 137)] while caused high DALY rates [585.4 (95% UI: 479.2, 701.3) and 761 (95% UI: 598.3, 957.3)], respectively. Similarly, 21 GBD regions showed decreasing or little fluctuation in incidence rate from 1990 to 2019, while most regions showed an increase from 2014 to 2019. For DALY rate, both 1990–2019 and 2014–2019 showed decreases except for high-income North America. In high-income North America, a rise in DALYs occurred in 2019 compared to 1990 [EAPC 2.6 (95% UI: 2.1, 3)] and a decline compared to 2014 [EAPC −2.5 (95% UI: −3.8, −1.2)]. Interestingly, from 2014 to 2019, East Asia showed a highest EAPC in incidence rate [9.1 (95% UI: 8.3, 9.9)], while DALY rate showed a large decrease [EAPC −7.6 (95% UI: −9.9, −5.4)]. And comparing the EAPC of incidence rate from 1990 to 2019, we found the contrast between trends of 1990–2014 and 2014–2019, with 1990–2014 showing a larger decline. Also worth noting is that Western Europe showed the highest incidence rate [326.6 (95% UI: 227.5, 480.3)] and the lowest DALY rate [85.8 (95% UI: 72.1, 101.1)] in 2019.

### 3.3. SDI

**Figure 3:**
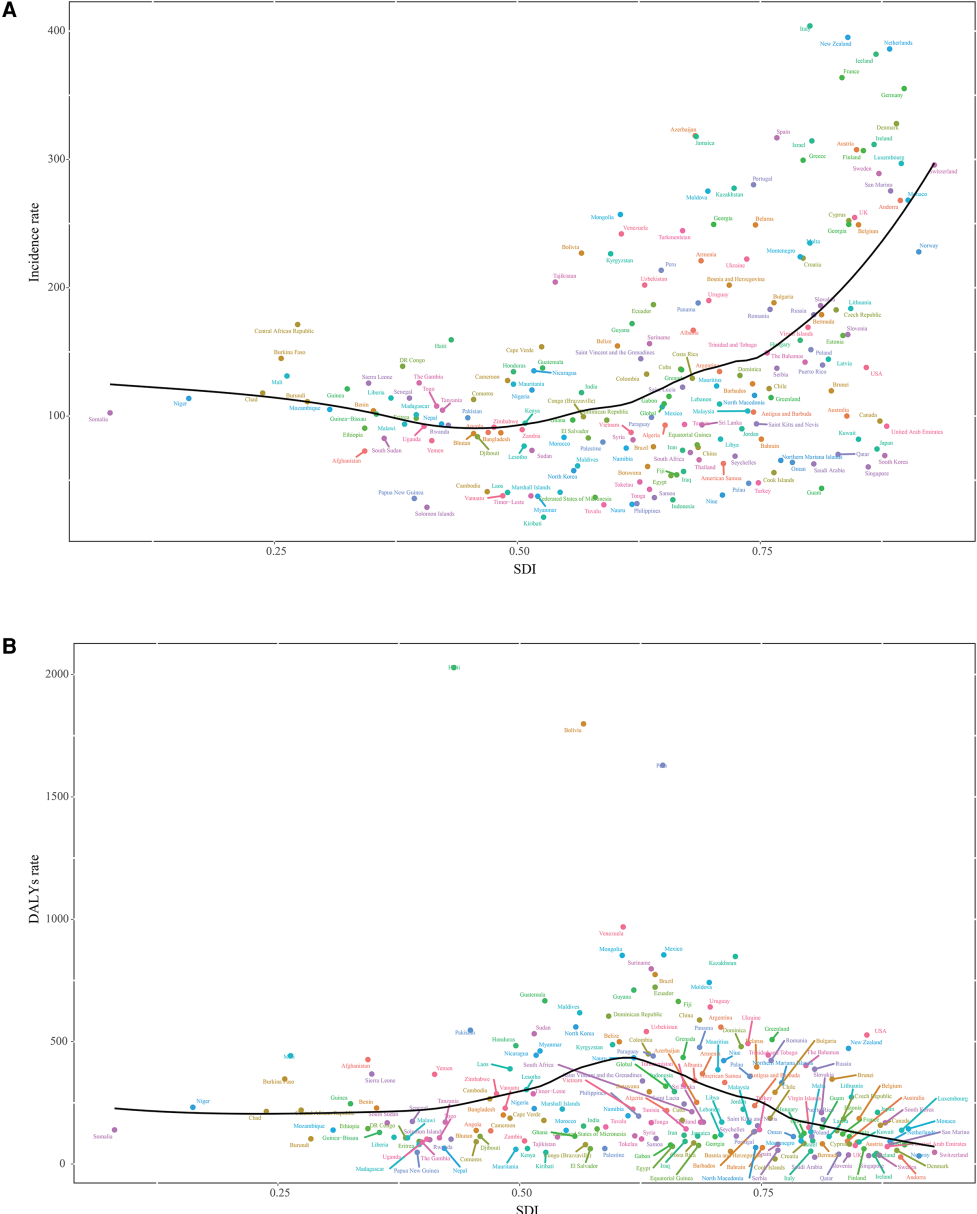
Association of incidence and DALY rates with socio-demographic index (SDI) for 204 countries and territories for foreign body aspiration among children under 5 years of age, in 2019. (**A**) Incidence rate; (**B**) DALY rate.

Although the incidence rates of low-middle, middle and high-middle SDI regions were lower than in high SDI region, the DALY rates were higher than in high SDI region in 2019. Particularly, in middle SDI region, the incidence rate showed the lowest but the age-standardized DALY rate showed the highest (Table 1). Of note, high SDI region exhibited significantly high incidence rate while low or middle SDI regions had low value in 2019. But for DALY rate, middle SDI countries instead showed a higher value (Figure 3). In 2014–2019, among the different SDI regions, incidence rate increased more in high SDI regions [EAPC 4.5 (95% UI: 3.6, 5.3)], and DALY rate had a smaller reduction compared with other regions [EAPC −2.7 (95% UI: −3.7, −1.6)]. Although DALY rate in the middle SDI region remained in the first place, there had been a large decline [EAPC −5.5 (95% UI: −6.7, −4.3)] (Table 1).

### 3.4. Age- and sex-specific patterns

In 2019, in different age groups, the post neonatal group showed the highest incidence rate [114 (95% UI: 70, 175.7)] and the early neonatal group showed the lowest incidence rate [92 (95% UI: 58.3, 153.6)], while the early neonatal group showed the highest DALY rate [8071.1 (95% UI: 6658.3, 9728.2)]. From 2014 to 2019, the incidence rate increased in every age group and the largest EAPC was observed in the age group 1 to 2 [2.4 (95% UI: 1.8, 3)]. And the DALY rate showed a decrease. In terms of gender, we did not find a significant difference in their incidence rates, DALY rates and EAPCs (Table 1).

## 4. Discussion

Although the incidence rate of FBA in children under five years of age declined significantly from 1990 to 2014, a rebound was observed from 2014 to 2019. In 2019, FBA among children under five years old caused a large incidence and DALYs globally. Different age groups and regions showed different profiles in terms of incidence and DALY rates. No significant differences were found between the sexes. From 1990 to 2019, incidence rates showed a decline in all age groups and most regions. Conversely, from 2014 to 2019, an increase in all age groups and most regions was observed. For the DALY rate, the trend remained down except for a few countries.

Foreign body aspiration occurs more often in childhood and remains a significant cause of death (5, 15). The causes and incidence rates of different age groups are different (11, 16–19). Airflow resistance is inversely proportional to the fourth power of the airway radius, so even small changes in the cross-section of a toddler's airway can result in dramatic changes in airway resistance and airflow (5). In our study, in 2019, the post-neonatal group had a higher incidence rate, while the incidence rate of the early neonatal group was lower, resulting in more DALY rate. This reminds us of the need to improve emergency and treatment methods and related policies, which may significantly reduce the DALY rate. It is also important to note that from 2014 to 2019, children in the 1–4 groups showed a higher EAPC of incidence rate compared to other age groups. All these findings have implications for targeting the appropriate strategies for different ages children.

Due to differences in economic status and policies in different SDI regions, the incidence and DALY rates of FBA varied among SDI regions (15). Although public health achievements have benefited high-income countries, citizens of low-middle-income countries have not enjoyed the same progress to a large extent (11). This also reflected the degree of attention to the disease and the effectiveness of related measures in each region. We found that in 2019, the incidence rate was higher in high SDI areas than in low-middle, middle, and high-middle SDI regions, but the DALY rate was lower than theirs. In general, the better economic areas have better medical conditions and are better able to take emergency measures in a timely manner, thus reducing DALYs (20, 21). Of note is that in middle SDI regions, perhaps because of the low reported incidence rate, it has instead reduced the attention to the disease, which led to a higher DALY rate than in other SDI regions in 2019. A study suggests that even if foreign body aspiration seem to frequently happen under adult surveillance, data regarding foreign body aspiration dynamics are never reported in case series collected in low-middle income countries (11). Thankfully, there was a significant decline in DALY rate compared to 2014. This demonstrated the effectiveness of the existing relevant policies and measures. The results of the analysis of the association of SDI with incidence and DALYs in 2019 also showed the same relationship as described above.

The 21 GBD regions also showed significant differences in incidence and DALYs. In 2019, Andean Latin America and Caribbean are in the top two for DALY rate and also show a high incidence. Although the incidence rate in East Asia and Tropical Latin America is not high, it results in a high DALY rate. As with the high SDI region described above, Western Europe showed the highest incidence rate and the lowest DALY rate. These were more reflective of the impact of economics, technology, and attention on the burden of disease. East Asia continued to have a higher DALYs than many other GBD regions in 2019, but showed a significant decline compared to 2014. This may be due to the improvement in economics and technology in recent years. However, it should not be ignored that although its incidence rate was not high in 2019, there is a large increase compared to 2014. In addition, most other GBD regions also showed an increasing trend in incidence from 2014 to 2019, although there were decreases or small fluctuations from 1990 to 2019. this suggests the need for improved and increased measures in prevention. Unlike other GBD regions, only High-income North America experienced a rise in DALY rate from 1990 to 2019, and thankfully, it occurred a decline from 2014 to 2019. At the national level, many European countries exhibited high incidence rates. However, it has been noted in the literature that there is no difference in prevalence between high-income and low-income countries. The higher incidence in high-income countries and high SDI regions in this study may be due to the poor surveillance and reporting system or the underdiagnosis of the disease in other areas (11, 22). The government has taken many effective measures, such as a publicly owned registry promoted by the European Union, the “Susy Safe Registry” (23). The high incidence rates had not resulted in a significant disease burden in these countries such as Italy, Netherlands, Iceland, France, and Germany. Conversely, Haiti, Mexico and Brazil did not have high incidence rates, but they ranked in the top 10 for DALY rate, especially Haiti, which ranked first. As in the middle SDI regions above, lack of sensitivity on the part of both parents and clinicians in identifying and acknowledging the risk (6, 9, 11). In addition, Bolivia (Plurinational State) and Peru had the second and third DALY rates, which may be influenced by the economy and technology of the country. It should also not be overlooked that the EAPCs of incidence rates in China, Netherlands, and Malta were in the top three and the DALY rates in Mali, Dominica, Puerto Rico, Lesotho, and Barbados showed an increase from 2014 to 2019.

Therefore, these countries and regions need to establish the necessary monitoring and reporting systems. Focus resources and prevention programs on at-risk populations, environments, and products identified through surveillance systems. Relevant institutions actively cooperate with the work, and open information sharing. Relevant organizations can work with manufacturers to assess foods and items that children may come into contact with and label them with warnings (11). Conduct anticipatory guidance and targeted education. Emphasize the importance of preventive measures and provide first aid instruction to parents, teachers, child care providers and others who care for children. Pediatricians and other infant and child health personnel to enhance prevention counseling and provide caregivers with information and guidance on safe behaviors (10, 11). To further improve cost-effectiveness, we also need to improve assessment and management techniques, such as flexible bronchoscopy, which has improved success rates and reduced complication rates in recent years. Bronchoscopy is the primary means of diagnosis and management, and its use requires increased physician expertise and experience (10, 19, 24, 25). In addition, we can distinguish patients who need intervention from those who can be safely observed and select appropriate patients for dynamic management (26, 27). To further reduce the burden of FBA on children under five years of age, it is necessary to understand the disease in different age groups and regions, and take into account the lifestyle and cultural habits of the population so that targeted policies and measures can be more effective.

This study also has some limitations. First, the GBD did not provide data on risk factors for FBA. We were unable to analyze which risk factors were dominant or the annual change in the contribution of risk factors. So, we can't offer some more focused advice. Second, due to differences in economic conditions, health expenditure, medical standards, and health policies, there exists heterogeneity in the epidemiological monitoring systems of different countries and regions, which may lead to some biases in the GBD reporting systems. We cannot rule out that the disease burden in some countries may be underestimated or overestimated. Third, following the existing age groupings of GBD, this study focused on children under 5 years of age. Further analysis may be needed for areas with high incidence in children of other ages. Despite these limitations, we hope that the present study will contribute to the development of policies and measures to reduce the disease burden of FBA in children.

## 5. Conclusion

In conclusion, the global incidence rate of FBA among children under 5 years old showed an increase in 2014–2019, although a decrease occurred from 1990 to 2014. For DALYs, it was a continuous downward trend from 1990 to 2019. The incidence and the DALY rates presented differently in different regions and age groups, and were correlated with SDI. Of concern is that the early neonatal group and middle SDI region have lower incidence rates, but they instead result in higher DALY rates than other age groups and regions. Therefore, relevant organizations and agencies need to improve the necessary monitoring and reporting systems, hazard assessment, enforcement, and public education activities.

## Data Availability

Publicly available datasets were analyzed in this study. This data can be found here: The Global Burden of Disease (GBD) Study 2019 (http://ghdx.healthdata.org).
