# Supplementary material for: Changes in the global burden of foreign body aspiration among under-5 children from 1990 to 2019
Source: Front Pediatr. 2023 Sep 1;11:1235308. doi: 10.3389/fped.2023.1235308 (PMC10506258; doi:10.3389/fped.2023.1235308)
Supplement: Supplementary file 2 [file Image1.pdf]

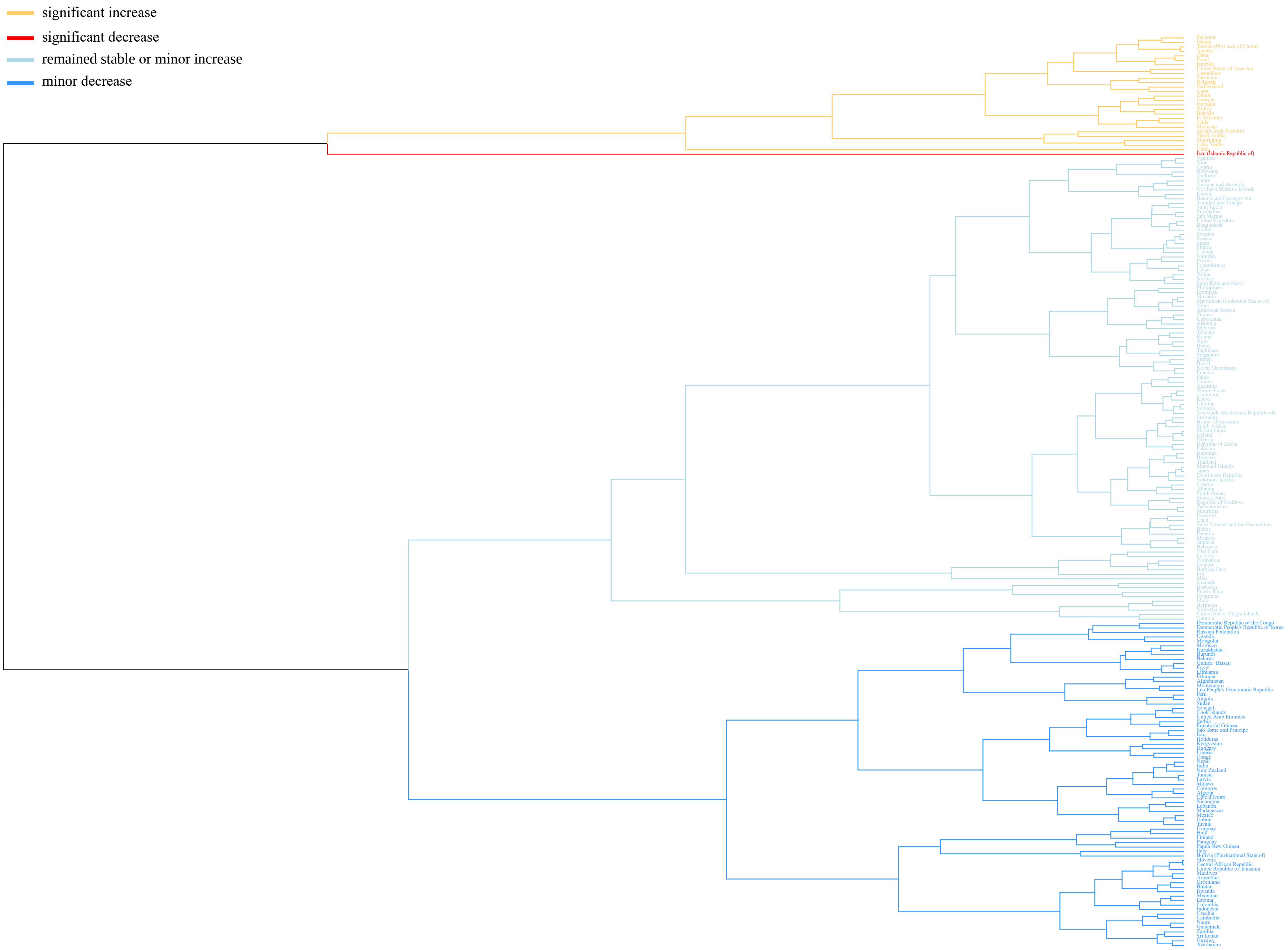

Figure S1 Hierarchical clustering of 204 countries and territories, by EAPC of incidence and DALY rates from 2014 to 2019.
